# Effects of Antibody Responses to Pre-Existing Coronaviruses on Disease Severity and Complement Activation in COVID-19 Patients

**DOI:** 10.3390/microorganisms10061191

**Published:** 2022-06-10

**Authors:** Massimo Cugno, Pier Luigi Meroni, Dario Consonni, Samantha Griffini, Elena Grovetti, Cristina Novembrino, Adriana Torri, Gloria Griffante, Marisa Gariglio, Luca Varani, Flora Peyvandi

**Affiliations:** 1 Department of Pathophysiology and Transplantation, Università degli Studi di Milano, 20122 Milan, Italy; flora.peyvandi@unimi.it; 2 UOC Medicina Generale–Emostasi e Trombosi, Department of Internal Medicine, Fondazione IRCCS Ca’ Granda, Ospedale Maggiore Policlinico, 20122 Milan, Italy; samantha.griffini74@gmail.com (S.G.); elena.grovetti@tiscali.it (E.G.); cristina.novembrino@policlinico.mi.it (C.N.); adriana.torri@policlinico.mi.it (A.T.); 3 Immunorheumatology Research Laboratory, IRCCS Istituto Auxologico Italiano, 20145 Milan, Italy; pierluigi.meroni@unimi.it; 4 Epidemiology Unit, Fondazione IRCCS Ca’ Granda, Ospedale Maggiore Policlinico, 20122 Milan, Italy; dario.consonni@unimi.it; 5 Virology Unit, Department of Translational Medicine, University of Piemonte Orientale, 28100 Novara, Italy; gloria.griffante@uniupo.it (G.G.); marisa.gariglio@med.uniupo.it (M.G.); 6 Institute for Research in Biomedicine, Università della Svizzera italiana (USI), 6500 Bellinzona, Switzerland; luca.varani@irb.usi.ch

**Keywords:** antibodies, COVID-19, SARS-CoV-2, OC43, HKU1, NL63, 229E, neutralisation assay, complement, endothelium, D-dimer, von Willebrand factor

## Abstract

The severity of coronavirus disease 2019 (COVID-19) may be influenced by pre-existing immune responses against endemic coronaviruses, but conflicting data have been reported. We studied 148 patients who were hospitalised because of a confirmed diagnosis of COVID-19, classified mild in 58, moderate in 44, and severe in 46. The controls were 27 healthy subjects. At admission, blood samples were collected for the measurement of biomarkers of disease severity and levels of the IgG against the receptor-binding domain (RBD) of severe acute respiratory syndrome coronavirus-2 (SARS-CoV-2) and pre-existing coronaviruses OC43, HKU1, NL63 and 229E. Higher levels of IgG antibodies against the RBD of pre-existing coronavirus (with the highest significance for anti-HKU1 IgG, *p* = 0.01) were found in patients with mild disease, compared with those with moderate or severe disease. Multivariable logistic regression confirmed the association of high levels of antibodies to pre-existing coronavirus with mild disease and showed their associations with low levels of the complement activation marker SC5b-9 (*p* range = 0.007–0.05). High levels of anti-NL63 antibodies were associated with low levels of the coagulation activation marker D-dimer (*p* = 0.04), while high levels of IgG against 229E were associated with low levels of the endothelial activation marker von Willebrand factor (*p* = 0.05). Anti-SARS-CoV-2-neutralising activity of plasma positively correlated with anti-SARS-CoV-2 IgG (r = 0.53, *p* = 0.04) and with anti-HKU1 IgG (r = 0.51, *p* = 0.05). In hospitalised patients with COVID-19, high levels of antibodies to pre-existing coronaviruses are associated with mild disease, suggesting that their measurement could be useful in predicting the severity of the disease.

## 1. Introduction

Infections due to severe acute respiratory syndrome coronavirus-2 (SARS-CoV-2) display different clinical manifestations ranging from asymptomatic conditions to minor upper airway manifestations or interstitial pneumonia, known as coronavirus disease 2019 (COVID-19) [1,2,3]. COVID-19 can also evolve into severe acute respiratory distress syndrome (ARDS) that can be life-threatening [1,2,4,5] and/or can be associated with a range of non-respiratory conditions affecting the heart, circulatory system, kidney, liver, and skin [6]. An exaggerated immune response triggered by SARS-CoV-2 is believed to be involved in the pathophysiology of COVID-19 [7] through the release of proinflammatory cytokines and activation of the complement system, blood coagulation, and endothelial cells [1,8,9,10,11]. Indeed, the activation of the defence biological systems has been demonstrated to parallel the severity and activity of the disease [12]. Genetic susceptibility to severe forms of COVID-19 has been associated with the chromosome 3 cluster rs11385942 variant [13], and this variant was also associated with higher complement activation in COVID-19 [14]. The severity of the disease may also be affected by pre-existing humoral responses against endemic coronaviruses, e.g., OC43, HKU1, NL63, and 229E, as these antibodies may recognise homologous epitopes in SARS-CoV-2 antigens [15,16,17]. OC43, HKU1, 229E, and NL63 are responsible for 15–30% of common colds in adults (for a review, see Liu et al. [18]). OC43, first isolated in 1967, is epidemic during winter and is usually associated with mild upper respiratory symptoms but may have neuroinvasive properties. HKU1, first isolated in Hong Kong in 2004, is distributed worldwide mainly during the spring-summer period; symptoms include rhinorrhoea, cough, sore throat, and fever. Coronavirus 229E was first isolated in 1966 and is believed to originate from African bats; it tends to be epidemic during winter with mild upper respiratory symptoms but may cause life-threatening lower respiratory tract infections in immunocompromised patients. NL63 was first isolated in the Netherlands in 2004, is associated with relatively mild symptoms, and its seasonality is not restricted to the winter in tropical and subtropical regions. Antibodies directed against pre-existing coronaviruses may cross-react with SARS-CoV-2 antigens, becoming protective if such antibodies are neutralising [19] but detrimental if they are sub- or non-neutralising, as it may occur in the case of antibody-dependent enhancement (ADE) of the disease [20]. To the best of our knowledge, the relationship between the levels of IgG against pre-existing coronaviruses and the activation markers of the complement system, blood coagulation, or endothelial cells has never been assessed in COVID-19 patients during the acute phase of infection.

With this background, we evaluated a cohort of patients with COVID-19 of different severity measuring the plasma levels of antibodies of the IgG class against the receptor-binding domain (RBD) of the pandemic coronavirus (SARS-CoV-2) and of the pre-existing endemic coronaviruses associated with the common cold (OC43, HKU1, 229E, and NL63). The levels of specific antibodies were analysed in relation to COVID-19 severity and markers of activation of complement, coagulation, and endothelial cells. In a selected group of patients, the anti-SARS-CoV-2-neutralising activity of plasma was also evaluated.

## 2. Materials and Methods

### 2.1. Patients

We studied 148 patients (87 males and 61 females; median age 63 years, range 26–92 years) who were admitted to our hospital between 1 March and 15 April 2020, as previously described [12]. All patients had a PCR-confirmed diagnosis of COVID-19 pneumonia: in mild cases, there was no need for oxygen therapy; moderate cases had oxygen saturation ≤92% on room air and required supplemental oxygen or non-invasive ventilation, severe cases needed intensive care with mechanical ventilation [11,12]. In total, 58 patients were considered to have mild disease, 44 had moderate, and 46 had severe disease. Demographic and clinical characteristics of patients with the relative haematochemical parameters of severity are reported in Table 1. In total, 27 healthy subjects served as controls (8 women and 19 men; median age 55 year, range 34–78 year). We collected blood samples at admission for the measurement of antibody levels of the IgG class against the RBD of SARS-CoV-2 and of the pre-existing coronaviruses OC43, HKU1, NL63, and 229E. We evaluated the relationships of the specific antibody levels with the levels of soluble C5b-9 (complement activation markers), D-dimer (coagulation activation marker) von Willebrand factor (vWF; endothelial activation marker). EDTA plasma was used for the measurement of antibodies and complement markers, whereas sodium citrate tubes were used for the measurement of coagulation and endothelial markers. The samples were rapidly centrifugated at 2000× *g* for 15 min, and the plasma was aliquoted and stored at −80 °C. The Ethics Committee of Milano Area 2 approved the study (Prot. No. 360_2020).

### 2.2. RBD Expression and Purification

Codon-optimised nucleotide sequences encoding RBDs of SARS-CoV-2, OC43, HKU1, NL63, and 229E, with C-terminal 8× HisTag, were synthesised and cloned into the mammalian expression vector pcDNA3.1(+) by Genscript. All proteins were produced via transient PEI transfection in Expi293 F cells (ThermoFisher, Waltham, MA, USA), purified from the cell supernatants by HiTrap Chelating HP (Cytiva). Samples were used fresh or after brief storage at −80 °C. The RBDs were extensively analysed to ensure functionality, stability, proper folding, lack of aggregation, and batch-to-batch reproducibility, with an array of biophysical and biochemical characterisation including binding assays to recombinant human ACE2 and anti-RBD human monoclonal antibodies; surface plasmon resonance (SPR); circular dichroism (CD); dynamic light scattering (DLS); size exclusion chromatography; SDS–PAGE; analytical ultracentrifugation (AUC) for selected RBDs [21].

### 2.3. Measurement of Anti-Coronavirus IgG Antibodies

Anti-coronavirus antibodies were assayed with an in-house ELISA that used the purified RBD of the various coronaviruses for capture and anti-human IgG for detection. Purified RBD of SARS-CoV-2, OC43, HKU1, NL63, and 229E was coated overnight onto microtitration plates (1 μg/mL in PBS, pH 7.4), and after washing, the wells were coated with BSA to avoid non-specific binding. After washing, 1:100 dilutions of plasma samples were added and incubated for 45 min at room temperature. After additional washes, the RBD-bound immunoglobulins were identified with monoclonal anti-human IgG-peroxidase-conjugated (Sigma-Aldrich, St. Louis, MO, USA) and revealed with orthophenylenediamine. The absorbance reading was made at 490 nm. The results were referred to internal standards and expressed as units per millilitre. Each internal standard was the plasma collected from a patient with a high anti-specific RBD antibody titre arbitrarily fixed at 100 units per millilitre.

### 2.4. Measurement of Activation Biomarkers

Plasma levels of soluble C5b-9 (SC5b-9) were measured as a marker of complement activation using a solid-phase assay (MicroVue Complement SC5b-9 Plus EIA kit, Quidel Corporation, San Diego, CA, USA) whose intra- and inter-assay coefficients of variation (CVs) were, respectively, 6.8% and 13.1%; the lower detection limit was 3.7 ng/mL.

D-dimer levels were measured as markers of coagulation activation via automated latex agglutination tests using anti-D-dimer monoclonal antibodies according to Kumano et al. [22]. The intra- and inter-assay coefficients of variation were lower than 3.8%.

Plasma levels of von Willebrand factor (vWF) antigen were measured as markers of endothelial activation using a commercial method (HemosIL Von Willebrand Factor Antigen, Instrumentation Laboratory, Bedford, MA, USA) with intra- and inter-assay CVs of <2.3%; the lower detection limit was 2.2%.

### 2.5. Anti-SARS-CoV-2-Specific Neutralising Antibody Assay

On a selected number of patients with severe COVID-19, the neutralisation assay was carried out as previously described [23,24]. Briefly, diluted EDTA plasma samples were incubated with the replication-competent vesicular stomatitis virus rVSV-SARS-CoV-2-SΔ21 (kindly provided by Dr. Sean P.J. Whelan, Washington University School of Medicine, USA) [24]) at a multiplicity of infection (MOI) of 0.05 for 1 h at 37 °C. Antibody–virus complexes were added to Vero E6-TMPRSS2 cells in 96-well plates and incubated at 37 °C for 24 h. Subsequently, cells were fixed in 4% formaldehyde (Millipore Sigma, St. Louis, MO, USA) containing 4′,6-diamidino-2-phenylindole (DAPI) for 15 min on ice. Images were acquired with the Leica THUNDER imager (Leica Microsystems, Wetzlar, Germany) in both the DAPI and fluorescein isothiocyanate (FITC) channels to visualise nuclei and infected cells (i.e., enhanced green fluorescent protein (eGFP)-positive cells), respectively (5 X objective, 9 fields per well, covering the entire well). Images were processed using the Leica Application Suite X (LAS X). A background number of eGFP+ cells were subtracted from each well using an average value determined from at least 2 uninfected wells. Data were processed using Prism software (GraphPad Prism 6.0), and the ID50 (the reciprocal dilution inhibiting 50% of the infection) was calculated by plotting and fitting the log of serum dilution versus response to a 4-parameter equation.

### 2.6. Statistical Analysis

Due to non-normal distribution, results were reported as medians and ranges (minimum to maximum) or percentiles, and non-parametric methods were used for comparison between groups. Categorical variables were reported as counts and percentages. Differences in proportions were assessed by using the chi-squared test. The associations between parameters were evaluated by logistic regression. Odds ratios (ORs) and 95% confidence intervals (CIs) were reported. Since hypertension, diabetes mellitus, and chronic obstructive pulmonary disease (COPD) are correlated to disease severity, we performed multivariable analyses adjusting for the variables simultaneously. The Spearman correlation coefficient was calculated to assess relationships between the variables. The data were analysed using the SPSS PC statistical package, version 27 (IBM SPSS Inc., Chicago, IL, USA), and the Stata 17 software (StataCorp. 2021, College Station, TX, USA).

## 3. Results

### 3.1. Relationships of COVID-19 Severity with Levels of Antibodies against RBD of SARS-CoV-2 or Pre-Existing Coronaviruses

**Figure 1:**
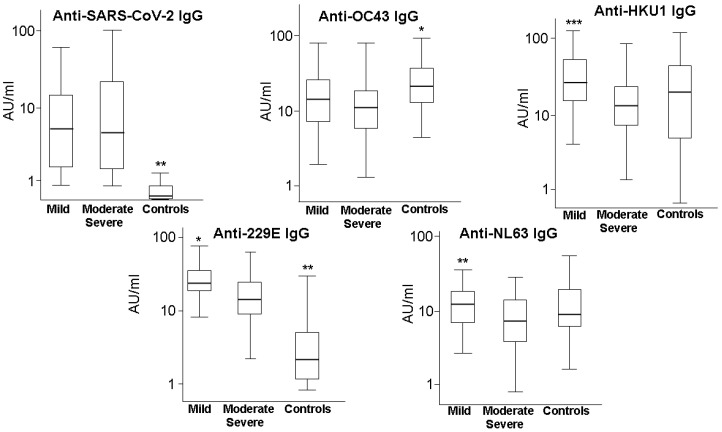
Plasma levels of antibodies of the IgG class directed against the receptor binding domain of SARS-CoV-2 and other coronaviruses causing common cold (OC43, HKU1, 229E, and NL63) in 148 patients with COVID-19 of different levels of severity. Boxes represent median, 25th and 75th percentile, while whiskers represent 5th and 95th percentile. Statistical significance of mild vs. moderate/severe: *** *p* = 0.006; ** *p* = 0.026; * *p* = 0.05. Statistical significance of controls vs. mild: ** *p* = 0.0001; * *p* = 0.048. Statistical significance of controls vs. moderate/severe: ** *p* = 0.0001; * *p* = 0.028.

No significant difference between patients with moderate disease and patients with severe disease was evident in levels of antibodies directed against the RBD of all the coronaviruses. Moreover, in multinomial (polytomous) logistic regression analyses, the ORs of moderate vs. mild disease and those of severe vs. mild disease were quite similar. For these reasons, and to simplify the presentation of results, we pooled data of patients with moderate disease, as well as those with severe disease, and fitted binomial logistic regression models. As shown in Figure 1, the levels of antibodies of the IgG class directed against the RBD of the coronavirus HKU1 were higher (*p* = 0.006) in patients with mild disease (median 23.99 AU/mL; range [3.23–108.33] AU/mL) than in patients with moderate or severe disease (median 13.78 AU/mL; range [0.63–72.35] AU/mL). In patients with mild disease, the levels of IgG against 229E (median 14.03 AU/mL; range [1.46–46.34] AU/mL) and the levels of IgG anti-NL63 (median 26.05 AU/mL; range [4.37–156.22] AU/mL) were also higher than those found in patients with moderate or severe disease (median 10.37 AU/mL; range [0.00–32.07] AU/mL for IgG anti-229E; median 18.02 AU/mL; range [2.42–105.33] AU/mL for IgG anti-NL63; *p* = 0.05 and *p* = 0.026, respectively) (Figure 1). No significant difference was evident in levels of antibodies anti-SARS-CoV-2 and anti-OC43 between patients with mild disease and patients with moderate or severe disease. Concerning the differences between hospitalised patients and controls in the levels of antibodies directed against the various coronaviruses, we found slightly higher levels of IgG anti-OC43 in the control group than in patients with mild disease (*p* = 0.048) or with moderate/severe disease (*p* = 0.028). No significant differences were evident between hospitalised patients and controls in the levels of antibodies against HKU1 and NL63, whereas controls had significantly lower levels of antibodies against SARS-CoV-2 and 229E (*p* = 0.0001) (Figure 1).

**Figure 2:**
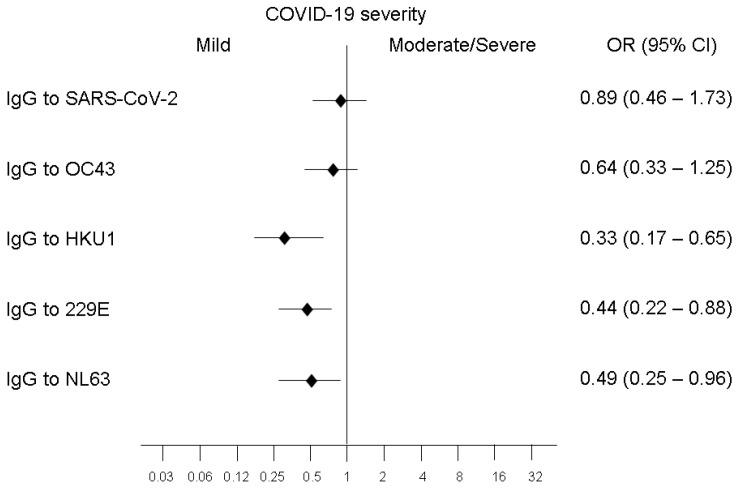
Association between high levels of specific IgG and the severity of the disease. Mild disease is associated with high levels (defined as levels higher than the median) of IgG against HKU1 (*p* = 0.001), 229E (*p* = 0.01) and NL63 (*p* = 0.018).

Logistic regression analysis (Figure 2) showed an association between mild disease and high levels (defined as levels higher than the median) of IgG against HKU1 (*p* = 0.001), 229E (*p* = 0.01) and NL63 (*p* = 0.018). Multivariable analysis considering comorbidities (hypertension, diabetes, and chronic obstructive pulmonary disease) confirmed the associations.

### 3.2. Association of Biomarkers of Complement, Coagulation, and Endothelium Activation with Antibodies to Coronaviruses

**Figure 3:**
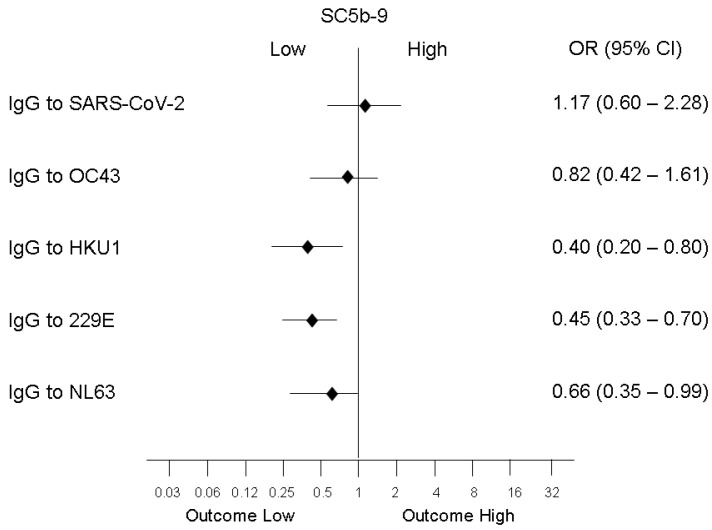
Association between high levels of specific IgG and normal levels of the complement activation marker SC5b-9. Normal levels of SC5b-9 are associated with high levels (defined as levels higher than the median) of IgG against HKU1 (*p* = 0.001), 229E (*p* = 0.01), and NL63 (*p* = 0.05).

Low levels of the complement activation marker SC5b-9 (within the normal range) were associated with high levels of IgG (above the median) against HKU1 (*p* = 0.001), 229E (*p* = 0.01), and NL63 (*p* = 0.05), while no significant association was evident with high levels of IgG against SARS-CoV-2 and OC43 (Figure 3). Low levels of the coagulation activation marker D-dimer were associated only with high levels of IgG to NL63 (OR 0.25; 95% CI [0.065–1.025]; *p* = 0.04) while low levels of the endothelial activation marker vWF were associated only with high levels of IgG to 229E (OR 0.15; 95% CI [0.018–1.29]; *p* = 0.05).

### 3.3. Correlation between Antibodies to SARS-CoV-2 and Antibodies to Pre-Existing Coronaviruses

Levels of IgG against the RBD of SARS-CoV-2 were positively correlated with levels of IgG against the RBDs of 229E (r = 0.48, *p* = 0.0001), HKU1 (r = 0.45, *p* = 0.001), and NL63 (r = 0.47, *p* = 0.0001). The weak direct correlation between antibodies against the new coronavirus and those against the previous ones may be consistent with a certain degree of cross-reactivity between current and previous coronaviruses.

### 3.4. Anti-SARS-CoV-2-Specific Neutralising Activity in 16 Patients with COVID-19

**Figure 4:**
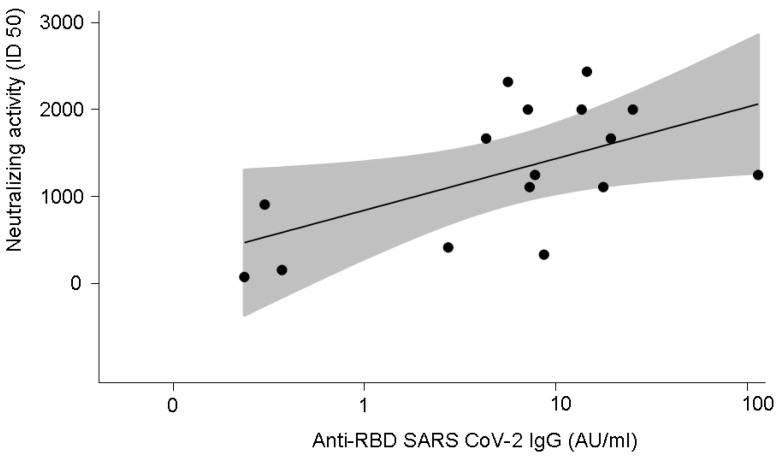
Correlation between SARS-CoV-2-neutralising activity and anti-SARS-CoV-2 antibody levels (r = 0.53, *p* = 0.04). The grey shadow represents the 95% confidence interval.

**Figure 5:**
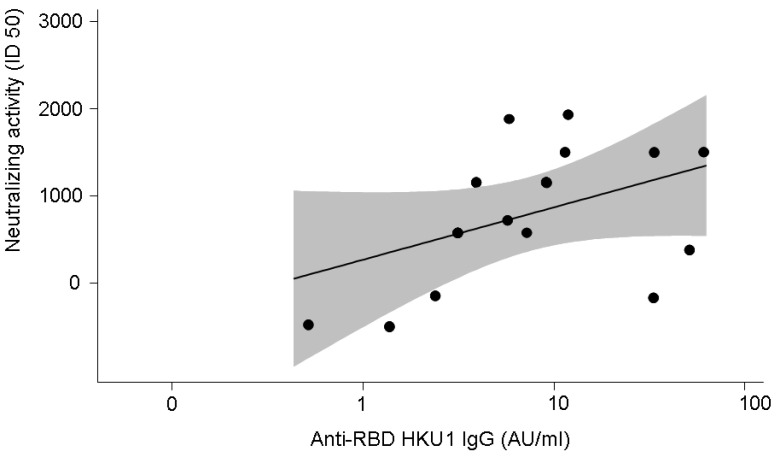
Correlation between SARS-CoV-2-neutralising activity and anti-HKU1 antibody levels (r = 0.51, *p* = 0.05). The grey shadow represents the 95% confidence interval.

Given the limited availability of samples, SARS-CoV-2-specific neutralising activity was performed in plasma from 16 patients with COVID-19 (M/F 11/5; age range 26–60 years, median age 60 years). The disease was severe in 11 patients and mild in 5. Neutralising activity positively correlated with anti-SARS-CoV-2 IgG (r = 0.53, *p* = 0.04) (Figure 4) and with anti-HKU1 IgG (r = 0.51, *p* = 0.05) (Figure 5).

## 4. Discussion

In this study, we found that the serum levels of IgG directed against pre-existing coronaviruses (HKU1, 229E, and NL63) were higher in patients with mild COVID-19 than in patients with moderate or severe disease. The logistic regression analysis of our data in 148 patients with COVID-19 showed an association of clinically mild disease with high levels of antibodies directed against previous coronavirus (HKU1, 229E, and NL63). Such antibodies were also associated with low levels of the complement activation marker SC5b-9, while the association with low levels of the coagulation marker D-dimer and of the endothelial marker vWF occurred only for NL63 and 229E, respectively. The positive correlation between serum-neutralising activity and anti-SARS-CoV-2 IgG or anti-HKU1 IgG observed in our COVID-19 patients is consistent with a response that is protective against aggravation. The slight direct correlation between antibodies directed against SARS-CoV-2 and those directed against pre-existing coronaviruses may indicate a cross-reactivity of the current antibodies with the spike proteins of the previous coronaviruses. The cross-reactivity with the actual coronavirus may involve humoral response as well as cellular immunity. Antibodies and cellular immunity may act in concert [25], thus contributing to the favourable effects of the previous immunisations. The pre-existing immune response may also counteract the dysregulation of complement induced by the SARS-CoV-2 spike protein. Indeed, it has been demonstrated that SARS-CoV-2 spike protein can promote the activation of the alternative pathway of complement by binding heparan sulphate on the endothelial cell surface [26], and its neutralisation, therefore, may eliminate a source of complement activation. Moreover, aggressive COVID-19 was shown to be associated with the deposition of immunoglobulins and products of activation of the classical complement pathway in the affected tissues [27]. The protective role of the cross-reactive antibodies that contrast SARS-CoV-2 may be also exerted by affecting the vicious loop between immune response and classical complement pathway activation.

Lin et al. showed that the antibodies against pre-existing coronaviruses may hinder SARS-CoV-2-effective immunity by studying 1202 individuals taken prior to SARS-CoV-2 infection and 107 COVID-19 patients. However, the authors recognised that a limitation of their study was the low number of participants requiring hospitalisation for severe (*n* = 2) or critical illness (*n* = 2) [28]. In contrast, our patients were all hospitalised, and high levels of antibodies against pre-existing coronaviruses were associated with mild disease. Our healthy controls showed slightly higher levels of antibodies against OC43 than hospitalised patients, in line with the hypothesis that antibodies against previous coronaviruses may be protective for SARS-CoV-2 infection; however, the wide overlap in the data of antibodies against OC43 and the lack of increase in the levels of antibodies against the other pre-existing coronaviruses in controls call for a cautious interpretation of the finding. Dugas et al., studying 296 COVID-19 patients, found lower levels of antibodies against OC43 nucleocapsid protein in patients with severe disease, concluding that prior infections with OC43 can protect against a severe course of COVID-19 [29]. In agreement with our results, Abela et al. found a protective effect of high levels of antibodies to pre-existing coronaviruses in 80 patients with COVID-19, of whom 16 were not hospitalised, 42 hospitalised in not intensive care unit, and 22 hospitalised in the intensive care unit [19]. Studying pre-pandemic and pandemic samples in SARS-CoV-2 infected patients, Anderson et al. found that humoral immune responses to pre-existing coronaviruses cross-react with SARS-CoV-2 but are not associated with protection against SARS-CoV-2 infection and do not affect the severity of COVID-19 [30], whereas Wratil et al. showed that pre-existing immunity to seasonal coronaviruses increases susceptibility to SARS-CoV-2 infection and COVID-19 severity [31]. In a retrospective study on 344 COVID-19 patients, Guo et al. found that disease severity correlates with levels of antibodies against OC43 spike protein, and no correlation was found with antibodies to the other pre-existing coronaviruses HKU1, 229E, and NL63 [32]. The mechanism hypothesised was the antibody-dependent enhancement of infection triggered by a non-protective antibody response to the coronavirus OC43, as reported in other coronavirus diseases [32]. These observations seem to contradict our data and those of Sagar et al., who reported that seasonal coronavirus infection is protective against severe COVID-19 outcomes, comparing 875 subjects with and 15,053 without a previously documented infection by endemic coronaviruses [33]. The explanation for the differences in these studies may lie in the different time courses between infections with pre-existing coronaviruses—the current SARS-CoV-2 infection and the decline in the immune response. Indeed, Sagar et al. showed that less-severe COVID-19 is associated with recent endemic coronavirus infections likely because of pre-existing and still active immune responses [33]. Garrido et al., by characterising anti-spike IgG responses in 28 non-hospitalised convalescent individuals across a spectrum of COVID-19 severity (13 with mild and 15 with severe disease) and 20 COVID-19 negative donors, demonstrated that humoral memory responses against seasonal coronaviruses contribute to COVID-19 disease severity, conferring either protection or risk, depending on epitope targeting [34].

Although our study has a sample size sufficient to ensure a reliable statistical power and alpha error, its main limitation is that it is a single-centre study from a single nation; thus, the generalisation of our message needs further confirmation. However, our data in well-characterised patients show that high levels of antibodies against pre-existing coronaviruses are associated with a favourable effect on COVID-19 and support the view that the serologic evaluation of previous infections with pre-existing coronaviruses may be useful in predicting the severity of COVID-19.

## Figures and Tables

**Table 1:** Demographic and clinical characteristics with the relative haematochemical parameters of severity in 148 patients hospitalised for COVID-19.

	Age Years	Sex M/F	Oxigen Need	D-Dimer μg/L	CRP mg/dL	Ferritin μg/L	Lymphocytes *n*/μL
**Mild** *n* = 58	61 (26–92)	35/26	No	810 (203–12,638)	5.35 (0.20–26.99)	483 (40–6384)	1170 (300–4550)
**Moderate** *n* = 44	64 (31–88)	28/16	Non-invasive ventilation	1038 (290–21,639)	7.40 (0.55–26.37)	1284 (69–8633)	915 (130–3330)
**Severe** *n* = 46	64 (27–90)	29/17	Mechanical ventilation	1667 (229–19,872)	10.95 (1.61–34.15)	1301 (206–11,366)	660 (180–2140)
**Normal ranges**				<500	0.00–0.05	30–400	1200–3400

Data are reported as median and ranges in parenthesis.

## Data Availability

The datasets generated and/or analysed during the current study are available from the corresponding author on reasonable request.
